# Re-evaluation of the impact of *BUD21* deletion on xylose utilization by *Saccharomyces cerevisiae*

**DOI:** 10.1016/j.mec.2023.e00218

**Published:** 2023-01-25

**Authors:** Venkatachalam Narayanan, Anders G. Sandström, Marie F. Gorwa-Grauslund

**Affiliations:** Division of Applied Microbiology, Department of Chemistry, Lund University, P.O. Box 124, SE-221 00, Lund, Sweden

**Keywords:** *Saccharomyces cerevisiae*, *BUD21*, Xylose, YPD vs. Defined medium, BY4741, CEN.PK 113-7D

## Abstract

Various rational metabolic engineering and random approaches have been applied to introduce and improve xylose utilization and ethanol productivity by *Saccharomyces cerevisiae*. Among them, the *BUD21* gene was identified as an interesting candidate for enhancing xylose consumption as its deletion appeared to be sufficient to improve growth, substrate utilization and ethanol productivity on xylose, even in a laboratory strain lacking a heterologous xylose pathway. The present study aimed at studying the influence of *BUD21* deletion in recombinant strains carrying heterologous oxido-reductive xylose utilization pathway. The positive effect of *BUD21* gene deletion on aerobic growth and xylose utilization could not be confirmed in two non-engineered laboratory strains (BY4741 and CEN.PK 113-7D) that were grown in YP rich medium with 20 g/L xylose as sole carbon source, despite the fact that effective deletion of *BUD21* gene was confirmed using both genotypic (colony PCR) and phenotypic (heat sensitive phenotype of the *BUD21* deletion mutant) control experiments. Therefore, the effect of *BUD21* deletion on xylose fermentation might be strain- or medium-dependent.

## Highlights

• The positive role of *BUD21* deletion was not confirmed in the tested strains/media.
• The impact of *BUD21* deletion on thermotolerance was confirmed.
• Defined media should be preferred to assess xylose utilization in *S. cerevisiae.*

## 1 Introduction

Sustainable ethanol production from lignocellulosic feedstock requires complete utilization of all the sugars that are composing cellulose and hemicellulose (Hahn-Hägerdal et al., 2006). This includes xylose that contributes to a substantial carbon portion in some lignocellulosic feedstocks, such as corn stover, hardwood or sugar cane bagasse (Hahn-Hägerdal et al., 2006). Therefore, *Saccharomyces cerevisiae,* that is the preferred organism for industrial bio-ethanol production, owing to its robustness towards industrially relevant fermentation conditions and ethanol titer (Rudolf et al., 2009), has been engineered for xylose utilization. Rational metabolic engineering and random approaches have been applied to introduce and improve xylose utilization and ethanol productivity by *S. cerevisiae* (recently reviewed by (Sun and Jin, 2021)). However, xylose utilization is still slow, as compared to glucose, so the search for targets leading to improvement of xylose fermentation rate continues. Several genes in endogenous *S. cerevisiae* metabolism have been shown to partially or completely suppress the utilization of xylose and hence were considered as deletion targets (Kim et al., 2013a). Some of the reported deletion targets which improved xylose metabolism include *YLR042c* (non-essential gene encoding a protein with unknown function localized in cell wall) (Parachin et al., 2010; Bengtsson et al., 2008), *MNI1* (encoding a putative S-adenosylmethionine-dependent methyltransferase), *RPA49* (encoding the *α*-subunit of RNA polymerase A) (Bengtsson et al., 2008), *ALD6* (encoding acetaldehyde dehydrogenase involved in acetate accumulation) (Kim et al., 2013b), *PHO13* (encoding an alkaline phosphatase involved in dephosphorylation of xylulose-5-phosphate) (Kim et al., 2013b; Ni et al., 2007), *IRA2* and *ISU1* (encoding Ras GTPase activating proteins involved in the cAMP/PKA pathway regulation and a mitochondrial Fe–S cluster scaffold protein, respectively) (Sato et al., 2016; Osiro et al., 2019) and *BUD21* (encoding a non-essential gene involved in ribosome biogenesis) (Usher et al., 2011).

*BUD21* (YOR078w) encodes a non-essential nucleolar component associated with U3 snoRNA (that is involved in pre-rRNA processing) required for ribosome biogenesis of small ribosomal subunit processome (SSU) (Shah et al., 2011; Dragon et al., 2002). *BUD21* was of particular interest as its deletion in wild type strains S288C and CEN.PK 113-13D was reported to enable xylose utilization, aerobic growth and ethanol production, even in the absence of introduced heterologous xylose utilization pathways (Usher et al., 2011). When a xylose utilization pathway (*XI, XKS*) was introduced in the *BUD21* deletion strain, aerobic fermentation with xylose (20 g/L) was reported to lead to increased amount of xylitol and reduced xylose consumption, which was attributed to a lower XI activity and/or increased expression of *GRE3* (Usher et al., 2011), *GRE3* being an endogenous aldose reductase gene capable of NADPH-dependent xylose reduction (Träff et al., 2001).

The aim of our study was to investigate the effect of *BUD21* gene deletion on xylose utilization in recombinant *S. cerevisiae* strains carrying, instead, a heterologous oxido-reductive xylose utilization pathway consisting of xylose reductase (*XR*), xylitol dehydrogenase (*XDH*) and further optimized by over-expression of gene encoding xylulokinase and several enzymes from the non-oxidative pentose phosphate pathway and deletion of the endogenous *GRE3* gene (Johansson and Hahn-Hägerdal, 2002). This could be a strategy to reduce xylitol yield thereby improving ethanol productivity when transformed to industrial *S. cerevisiae* strains. However, we show here that the initial evaluation of *BUD21* deletion in background strains led us to reconsider its importance for xylose utilization.

## 2 Materials and methods

### 2.1 General medium recipes and chemicals

Unless otherwise mentioned, all antibiotics, sugars and standards were purchased from Sigma-Aldrich (St. Louis, MO, USA), and culture medium components were obtained from BD Diagnostics (Franklin Lakes, NJ, USA). All strains were maintained in YPD agar plates containing 10 g/L yeast extract, 20 g/L peptone, 20 g/L glucose and 20 g/L agar, and stored at −80 °C in YPD medium containing 10 g/L yeast extract, 20 g/L peptone and 20 g/L glucose supplemented with 30% (v/v) glycerol. Growth experiments were carried out in YPG medium (10 g/L yeast extract, 20 g/L peptone, 20 g/L glucose), YPX medium (10 g/L yeast extract, 20 g/L peptone, 20 g/L xylose), YNG medium (Yeast Nitrogen Base without amino acids, 20 g/L glucose, 76 mg/L histidine, 380 mg/L leucine, 76 mg/L methionine, 76 mg/L uracil, potassium hydrogen phthalate (50 mM), pH 5.5) or YNX medium (Yeast Nitrogen Base without amino acids, 20 g/L xylose, 76 mg/L histidine, 380 mg/L leucine, 76 mg/L methionine, 76 mg/L uracil, potassium hydrogen phthalate (50 mM), pH 5.5).

### 2.2 Generation of deletion strains

Plasmids and strains used in this study are listed in Table 1. *BUD21* was deleted in the laboratory strain background CEN.PK 113-7D (Entian and Kötter, 2007) using one-step gene disruption strategy by a fragment generated by overlap-extension PCR. The gene encoding *KanMX4* that confers resistance to the antibiotic geneticin (G418) was chosen as selective marker (Güldener et al., 1996). The upstream (US) and downstream (DS) of *BUD21* gene were PCR amplified individually with primers BUD21_US_f, BUD21_US_r and BUD21_DS_f, BUD21_DS_r respectively (Table 2) and the PCR products were annealed to the flanking ends of the *KanMX4* gene from plasmid pUG6 (Güldener et al., 1996) by overlap-extension PCR utilizing Phusion Hot Start II High-Fidelity DNA Polymerase (Thermo Scientific, Waltham, MA, USA). The resulting fragment (BUD21-US-loxP-KanMX-loxP-BUD21-DS) was purified by QIAquick gel extraction kit and used for transformation. The fragment was transformed into CEN.PK 113-7D strain using the high efficiency LiAc method (Gietz and Schiestl, 2007) and the transformants were selected in YPD plates (10 g/L yeast extract, 20 g/L peptone, 20 g/L glucose and 20 g/L agar) supplemented with 200 mg/L geneticin (G418 sulfate) (Gibco, Thermo Scientific, Waltham, MA, USA). *BUD21* deletion was confirmed by PCR for at least 3 colonies and with 3 different sets of primers: 1) BUD21_US_f and KanMX_mid_r, 2) KanMX_mid_f and BUD21_DS_r and 3) BUD21_US_f and BUD21_DS_r (Table 2). Deletion of *BUD21* gene in ΔBY4741 strain background was verified using PCR with 3 different sets of primers: 1) BUD21_US_600bp_f and KanMX_mid_r, 2) KanMX_mid_f and BUD21_DS_600bp_r and 3) BUD21_US_f and BUD21_DS_r (Table 2). The *BUD21-*deleted strains were designated as ΔBY and ΔCEN.PK (Table 1).

**Table 1:** Strains and plasmids used in the study.

Strains & Plasmids	Abbreviation	Relevant genotype	References
pUG6 plasmid		KanR (loxP-KanMX4-loxP)	Güldener et al. (1996)
BY4741	BY	*MAT*a; *his3Δ1; leu2Δ0; met15Δ0; ura3Δ0*	Baker Brachmann et al. (1998)
BY4741 (*bud21Δ*)	ΔBY	BY4741; *MAT*a; *his3Δ1; leu2Δ0*; *met15Δ0; ura3Δ0*; YOR078w::*KanMX4*	EUROSCARF (Accession no.: COMP-SET3-A)
CEN.PK 113-7D	CEN.PK	*MAT*α, MAL2-8c, *SUC2*	Entian and Kötter (2007)
CEN.PK 113-7D (*bud21Δ*)	ΔCEN.PK	CEN.PK 113-7D, YOR078w::*kanMX4*	This study
TMB 3001		CEN.PK113-7A (MATa his3-D1 MAL2-8C SUC2) with *XYL1, XYL2* and overexpressed *XKS1*	Eliasson et al. (2000)

**Table 2:** Primers used in the study.

Amplification Target	Primer	Nucleotide sequence (5'→ 3′)
*BUD21* upstream	BUD21_US_f	CTCAATCGTGCTAAGCGACAAACTGAAAAG
	BUD21_US_r	GTTGTCGACCTGCAGCGTACGAAGCTGTCTTACTTAATACGTGTATACGGGTAGTAAC
*BUD21* downstream	BUD21_DS_f	GGTGATATCAGATCCACTAGTGGCCTATGCGGCCGTATATAGCATACAACATAATAAAATAATAGTACAATCAC
	BUD21_DS_r	GGAAATCTTCTGCTTATGAGTGGTGGCAG
KanMX from pUG6 plasmid	KanMX_f	CCGTATACACGTATTAAGTAAGACAGCTTCGTACGCTGCAGGTCGAC
	KanMX_r	GTGATTGTACTATTATTTTATTATGTTGTATGCTATATACGGCCGCATAGGCCACTAGTGG
*BUD21* 600bp Upstream and 600bp downstream	BUD21_US 600bp_f	AGCAGCGGCACGGAGAAAAATGGATTGATG
	BUD21_DS 600bp_r	GCATTAGGTACGACAACGAACAATGATTCACTGCTC
KanMX from Middle	KanMX_mid_f	GCATTAGGTACGACAACGAACAATGATTCACTGCTC
	KanMX_mid_r	GACGAAATACGCGATCGCTGTTAAAAGGAC

### 2.3 Aerobic growth assays

Growth assay comparisons was carried out between the following *S. cerevisiae* strains 1) BY (BY4741) and ΔBY (BY4741 strain with *BUD21* gene deleted (Accession number: COMP-SET3-A from EUROSCARF deletion mutant library), 2) CEN.PK (wild type CEN.PK strain 113-7D), ΔCEN.PK (CEN.PK 113-7D with *BUD21* gene deleted) and TMB3001, a genetically modified xylose-utilizing strain derived from CEN.PK113-7A (Table 1). Pre-inoculum was cultured from single colony of respective strain in 5 mL of YPG medium in 50 mL conical tube incubated for 15–20 h. Based on subsequent cultivation conditions (YPG/YPX media), the CEN.PK, ΔCEN.PK and TMB 3001 strains had an intermediate step where cells were transferred into an inoculum culture of 10 mL YPG/YPX medium with an initial OD of 0.2 and were incubated for 24 h. The cells from pre-cultures (BY and ΔBY strains) or intermediate cultures (CEN.PK, ΔCEN.PK and TMB3001 strains) were centrifuged at 2050×*g* for 5 min at 20 °C, washed with 5 mL of 0.9% sterile NaCl solution and the harvested cells were re-inoculated into 25 mL of YPG/YPX media or YNG/YNX media (for BY and ΔBY strains) in 250 mL baffled Erlenmeyer shake flasks with an initial OD of 0.2. Aerobic growth was performed at 30 °C in a rotary shake incubator (New Brunswick, Enfield, CT, USA) at 180 rpm. Absorbance measurements were performed at 620 nm to determine cell growth on a given sample diluted to an optical density (OD) below 0.3 (Spectrophotometer U-1800, Hitachi, Berkshire, UK).

### 2.4 Heat sensitivity assay

In order to confirm the phenotypic difference between CEN.PK and ΔCEN.PK, a growth curve was performed in YPG medium at 37 °C. Pre-inoculum was cultured from single colony of respective strains in 5 mL of YPG medium in 50 mL conical tube incubated for 18 h at 30 °C. The cells from these pre-cultures were re-inoculated into 25 mL of YPG medium in 250 mL baffled Erlenmeyer shake flasks with an initial OD of 0.2 and grown at 37 °C and 180 rpm.

All the aerobic cultivations were performed at least in biological duplicates. Student T-test was performed with two tailed distribution and two sample unequal variance using Microsoft excel 2013 software.

### 2.5 Metabolite analysis

Samples were withdrawn for metabolite analysis, quickly centrifuged and the supernatant was filtered through 0.2 μm membrane filters (Toyo Roshi Kaish, Tokyo Japan) and stored at −20 °C until analysis. Concentrations of glucose, xylose, glycerol, acetate and ethanol were determined by High Performance Liquid Chromatography (HPLC) (Waters, Milford, MA, USA) using a HPX-87H resin-based column (Bio-Rad, Hercules, CA, USA) preceded by a Micro-Guard Cation-H guard column (Bio-Rad, Hercules, CA, USA). Separation was performed at 45 °C and at flow rate of 0.6 mL min^−1^ with 5 mM H_2_SO_4_ as mobile phase. All compounds were quantified by refractive index detection (Shimadzu, Kyoto, Japan). For each HPLC run, a seven-point calibration curve was made for each compound to calculate concentrations. Each sample was analysed at least in duplicate.

## 3 Results and discussion

Before investigating the effect of *BUD21* gene deletion on xylose utilization in strains carrying the *Scheffersomyces stipitis* oxidoreductive xylose pathway, a preliminary experiment was performed to confirm the previously observed response of a non-engineered laboratory strains to *BUD21* gene deletion, i.e. the ability to grow on xylose without added heterogeneous xylose genes (Usher et al., 2011). Aerobic growth on xylose of the *BUD21* deletion mutant from EUROSCARF collection and the parental strain BY4741 (Table 1) were first compared in YPX medium. As *BUD21* deletion was also reported to improve growth on glucose (Usher et al., 2011), the comparison was also performed in YPG medium. In parallel, proper deletion of *BUD21* gene in the ΔBY strain background was confirmed using PCR (data not shown), which ruled out potential strain construction issues.

**Fig. 1:**
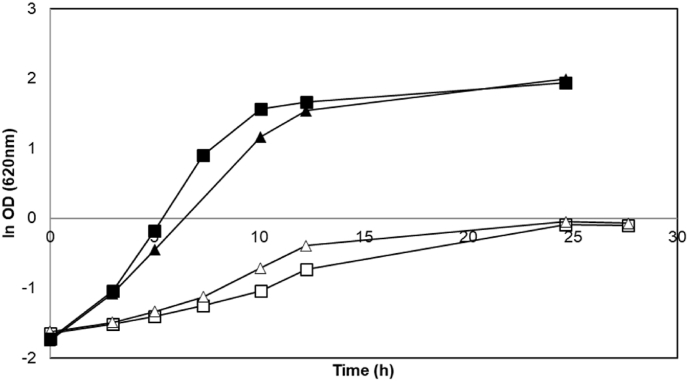
Representative aerobic growth of *S. cerevisiae* strains BY (squares) and ΔBY (triangles) in YPG (filled symbols) and YPX media (empty symbols). Experiments were performed in duplicates and less than 5% deviation was recorded.

Deletion of *BUD21* led to a marginal improvement in the maximum specific growth rate on xylose (Table 3, Fig. 1) as compared with wild type BY strain (P-value = 0.02); however, the effect was not as significant as previously reported (Usher et al., 2011) and the final OD values were similar (Table 3). When grown in glucose medium (YPG), ΔBY strain had a similar final OD compared to the wild type BY strain (P-value = 0.19) but a lower maximum specific growth rate than in wild type strain (P-value = 0.0001) (Table 3).

**Table 3:** Maximum specific growth rate (h^−1^) and final OD_620nm_ for *S. cerevisiae* strains grown aerobically at 30 °C in YP medium with 20 g/L glucose or xylose. Reported values are from biological replicates.

Strain	YP Glucose		YP Xylose	
	Growth rate	Final OD	Growth rate	Final OD
BY	0.451 ± 0.001	6.91 ± 0.09	0.094 ± 0.003	0.89 ± 0.01
ΔBY	0.316 ± 0.001	7.58 ± 0.32	0.132 ± 0.001	0.93 ± 0.00
CEN.PK	0.462 ± 0.002	5.35 ± 0.11	0.179 ± 0.000	1.73 ± 0.07
ΔCEN.PK	0.368 ± 0.003	5.75 ± 0.06	0.173 ± 0.002	1.64 ± 0.01
TMB 3001	0.391 ± 0.004	12.12 ± 0.21	0.248 ± 0.005	14.00 ± 1.34

**Fig. 2:**
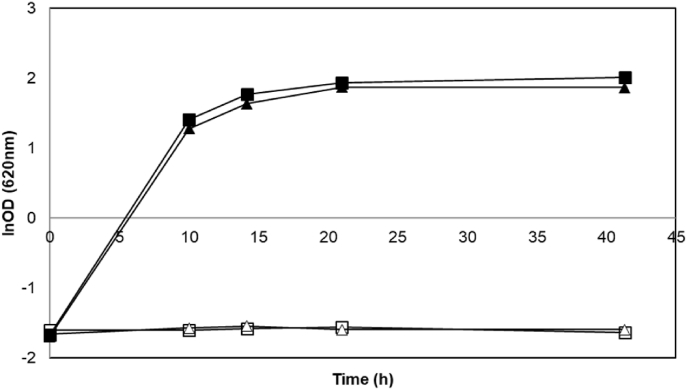
Representative aerobic of *S. cerevisiae* strains BY (squares) and ΔBY (triangles) in YNG (filled symbols) and YNX media (empty symbols). Experiments were performed in duplicates and with less than 5% deviation was recorded.

Yeast extract that is used in culture media may contain traces of glucose obtained, for instance, from glycogen and trehalose hydrolysis as well as amino acids and other biosynthetic precursors that are essential for anabolic pathways, thereby saving metabolic energy which can translate into improvement in growth (Hahn-Hägerdal et al., 2005). As the growth rate and final OD were poor but significant in YPX, we investigated the impact of the absence of yeast extract on the strain performances. BY and ΔBY strains were grown aerobically in defined mineral medium with glucose (YNG) or xylose (YNX) as unique carbon sources. As expected, the growth rate was lower in YNG than in the YPG medium for both strains. But, most important, similar growth profiles were obtained for BY and ΔBY strains in YNG medium, whereas no growth was observed for both strains in YNX medium (Fig. 2). This indicates that the marginal growth of BY and ΔBY strains in YPX medium might have not resulted from the use of xylose as carbon source and it supports previous work where growth of *S. cerevisiae* was observed in yeast extract medium lacking any added carbon source (Hahn-Hägerdal et al., 2005).

**Fig. 3:**
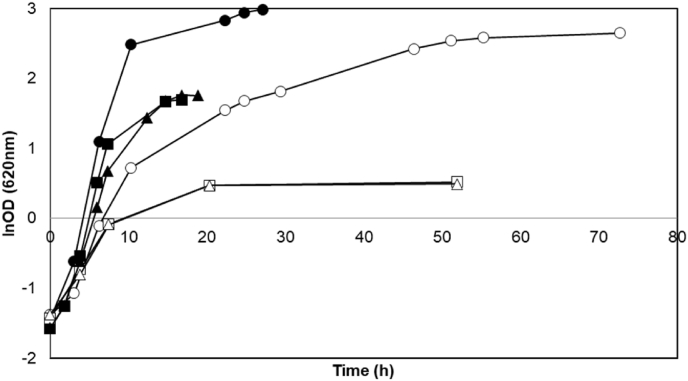
Representative aerobic growth of *S. cerevisiae* strains CEN.PK (squares), ΔCEN.PK (triangles) and TMB 3001 (circles) in YPG (filled symbols) and YPX media (empty symbols). Experiments were performed in duplicates and less than 5% deviation was recorded.

**Fig. 4:**
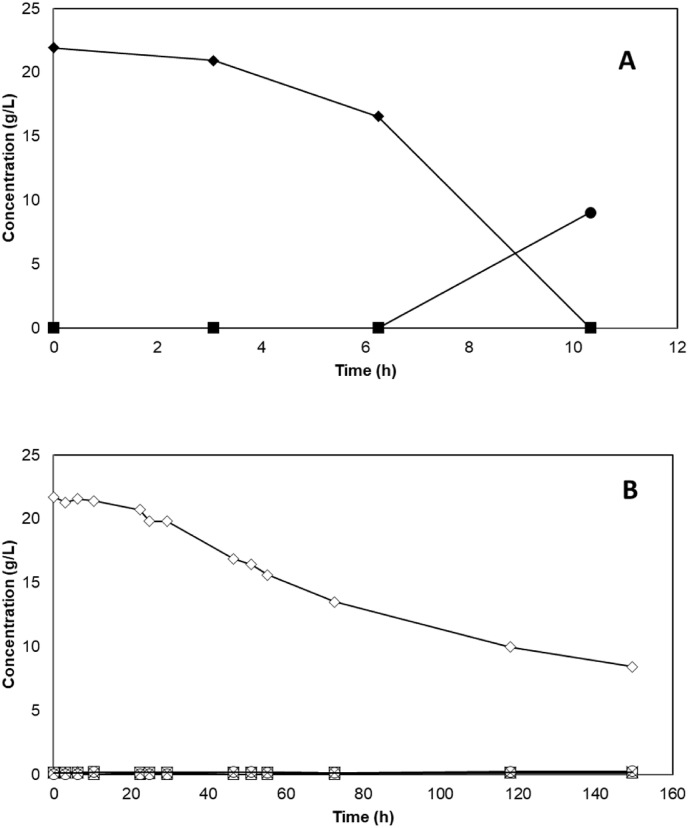
Fermentation profile of aerobic growth of *S. cerevisiae* strain TMB 3001 in YPG medium (A; filled symbols) and YPX medium (B; open symbols). Legend: glucose/xylose (diamond), glycerol (squares), acetate (triangle), ethanol (circle) and xylitol (cross). Experiments were performed in duplicates with less than 5% deviation.

Since this first result was unexpected, it was decided to delete *BUD21* in another strain background in order to evaluate whether *BUD21*-associated improvement could be strain specific. CEN.PK 113-7D (CEN.PK) was chosen to re-analyze the effect of *BUD21* deletion on xylose metabolism since it is a popular strain for physiological studies (van Dijken et al., 2000) and it is the same background strain as for the previous *BUD21* deletion study (Usher et al., 2011). A *BUD21*-deletion fragment was generated by overlap-extension PCR, and transformed into CEN.PK 113-7D and the successful deletion was confirmed using colony PCR towards the gene locus (data not shown). The *BUD21* deleted strain (ΔCEN.PK) was grown aerobically in YPX medium with the parental strain CEN.PK as control strain and YPG as control medium. The fermentation metabolite data indicated that xylose consumption was below detection limits (data not shown) in both CEN.PK and ΔCEN.PK strains, which demonstrated the absence of xylose utilization by both strains. This was also confirmed by the limited and identical growth profile in both strains (Fig. 3), that is expected to arise from the YP component of the medium, as discussed above. In order to validate the suitability of the used medium for growth on xylose, the xylose-utilizing control strain TMB 3001 (Eliasson et al., 2000) was cultivated in both YPG and YPX media. As expected, TMB 3001 grew in both media (Fig. 3) and used glucose in YPG (Fig. 4A) and xylose in YPX (Fig. 4B), with consumption rates of 2.94 ± 0.00 g L^−1^.h^−1^ and 0.09 ± 0.01 g L^−1^.h^−1^ on YPG and YPX, respectively; In contrast, no xylose consumption was measured in YPX medium for the *BUD21* deleted version of CEN.PK 113-7D strain (Fig. 4B).

**Fig. 5:**
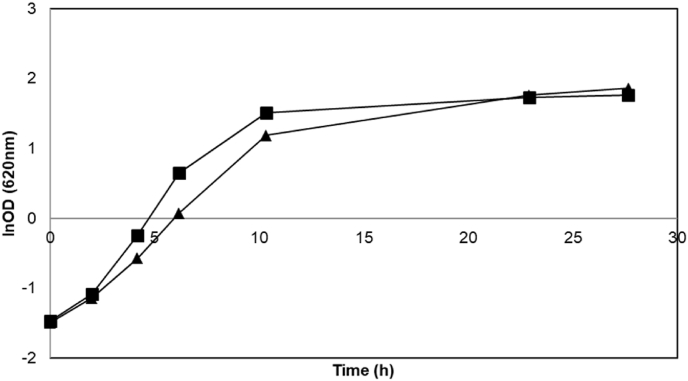
Representative aerobic growth of *S. cerevisiae* strains CEN.PK (squares) and ΔCEN.PK (triangles) in YPG at 37 °C. Experiments were performed in duplicates with less than 5% deviation.

As a last control to verify the correct inactivation of *BUD21* gene, CEN.PK and ΔCEN.PK strains were grown in YPG medium at 37 °C as the deletion of *BUD2*1 has been reported to decrease heat tolerance at elevated temperature (37 °C) (Sinha et al., 2008). Maximum specific growth rate of the ΔCEN.PK strain (0.28 h^−1^) indeed decreased 1.5 fold as compared with the CEN.PK strain (0.43 h^−1^) (P-value = 0.022) (Fig. 5), which confirmed the deletion phenotype. Consequently, it was not considered relevant to perform further deletion of *BUD21* in recombinant strains carrying the oxido-reductive xylose pathway.

*BUD21* is a 645 nucleotide ORF coding for a protein of 214 amino acids involved in ribosomal biogenesis processes, including production of 18S RNA, rRNA processing and 40S ribosome subunit assembly (Dragon et al., 2002; Granneman and Baserga, 2004). As ribosome biosynthesis is an extremely energy demanding process in rapidly growing cells (Shah et al., 2011), optimization or deletion of some non-essential ribosomal biogenesis pathway genes, that would reduce ribosomal protein expression, could be a strategy to grow and ferment *S. cerevisiae* strains under specific stress conditions that require the mobilisation of cell resources (Shah et al., 2011). This way, more resources and energy could be conserved and channelled to cell growth and tolerance during episodes of stress. It was indeed proved that *BUD21* gene mutation led to increased tolerance to hypoxia (Shah et al., 2011) and we could confirm the growth sensitivity at elevated temperature. Nevertheless, we could not corroborate that *BUD21* gene was involved in the initiation and improvement of xylose utilization from our aerobic growth studies.

Our results highlight the importance of the choice of the medium for the assessment of genetic manipulations. It was previously shown that baker's yeast can grow aerobically by utilizing yeast extract as the sole carbon source, with a maximum specific growth rate of 0.29 h^−1^ and final OD of 3–4 (Hahn-Hägerdal et al., 2005). It is therefore possible that the growth previously observed for *BUD21* deletion strains in YPX medium originated from the YP components and not from xylose itself. We have recurrently observed that different YP batches may contain different levels of remaining sugars, which impacts the interpretation of growth data. The study re-inforce the fact that comparison studies should be performed in defined mineral media, such as the one reported by (van Dijken et al., 2000).

Since there were slight difference in growth in YPX medium between strains of BY4741 and CEN.PK 113-7D, it is possible that strain background also plays a role in the growth phenotype. For instance, the improvement of xylose utilization was previously achieved by deleting *YLR042c* in different laboratory strain backgrounds but the effect was shown to be the most significant in the strain having the poorest growth rate (Parachin et al., 2010; Bengtsson et al., 2008). The same phenomenon might be applicable here with *BUD21* and the improvement of growth, which we attribute to the residual carbon source that is present in yeast extract, might be significant in terms of fold change but limited in terms of achieved biomass as well as mostly visible in strains having poor growth capacity.

## 4 Conclusions

*BUD21* gene deletion was previously reported to enable aerobic growth and xylose utilization in *S. cerevisiae* (Usher et al., 2011). This phenotype could not be reproduced in two different non-engineered laboratory strain backgrounds (BY4741 and CEN.PK 113-7D) and on different media, despite the fact that effective deletion of *BUD21* gene was confirmed. Therefore, the effect of *BUD21* deletion on xylose fermentation might be strain- or medium-dependent.

## Conflict of interests

The authors declare that they have no conflict of interests.

## Author statement

**Venkatachalan Narayanan**: Methodology, Investigation, Writing- Original Draft; **Anders Sandström**: Methodology, Writing-Review & editing; **Marie Gorwa Grauslund**: Conceptualization, Supervision, Writing-Review & editing, Funding acquisition.

## Declaration of competing interest

The authors declare that they have no known competing financial interests or personal relationships that could have appeared to influence the work reported in this paper.

## Data Availability

Data will be made available on request.
